# Supplementary material for: Reduced auditory steady state responses in autism spectrum disorder
Source: Mol Autism. 2020 Jul 1;11:56. doi: 10.1186/s13229-020-00357-y (PMC7329477; doi:10.1186/s13229-020-00357-y)
Supplement: Supplementary file 1 — Additional file 1: Supporting Figure 1: Regions of interest were defined in left and right Primary Auditory Cortex, according to HCP-MMP 1.0 Atlas. Supporting Figure 2: Sensor-level Analysis. (A) Group average topo-plot for the auditory M100 event-related field, magnetometers shown. (B) Group average topoplot for auditory steady state responses (ASSR) at 40Hz (C) Group average topoplot of the transient gamma-band response (tGBR), 30-60Hz, 0.0-0.1s. Scales represent MEG field strength, baseline-corrected, with units of Tesla/cm. Supporting Figure 3: Whole-brain maps showing changes in ASSR power, corresponding to Figure 2 in the main text. Supporting Figure 4: Responses to the Glasgow Sensory Questionnaire were grouped by sensory domain (maximum score = 20) and hypo- / hyper-sensitivity (green and blue bars respectively). Our data show a heterogeneous pattern of sensory symptoms, with mixture of hypo- and hyper-sensitivities. Auditory symptoms scored 13.9/20 corresponding to questionnaire answers closest to “Sometimes”. Supporting Figure 5: Scatter plots to show the relationship between ASSR power, averaged across left/right A1, and Glasgow Sensory Questionnaire (GSQ) Scores, summed across the six auditory questions only. There were no significant (p>.05) correlations for ASSR power, r = -.07, p=.77, or for ITC Z-Value (left A1), r = -.29, p=.24, (right A1), r = -.07, p = .76. The shaded region indicates 95% confidence intervals. ITC = Inter-trial Coherence; ASSR = Auditory Steady State Response. Supporting Figure 6: For ASSR power (top) and ITC power (bottom), the group average for the ASD group was subtracted from the group average for controls (at each time bin) and plotted for each ROI (right A1: red; left A1: green). The black dotted lines link the times maximum ITC group differences for left and right A1 with the corresponding data for ASSR power. Supporting Table 1: Results of the meta-analytical decoding (top 10 terms shown). The correlation term corresponds [file 13229_2020_357_MOESM1_ESM.pdf]

Supporting Information:

Reduced Auditory Steady State Responses in Autism Spectrum Disorder

Seymour, R.A., Rippon, G., Gooding-Williams, G,. Sowman, P.F. & Kessler, K.

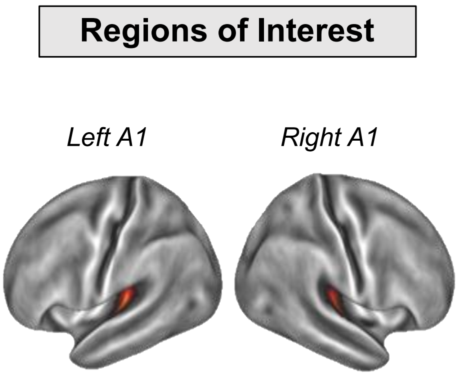

Supporting Figure 1: Regions of interest were defined in left and right Primary Auditory Cortex, according to HCP-MMP 1.0 Atlas.

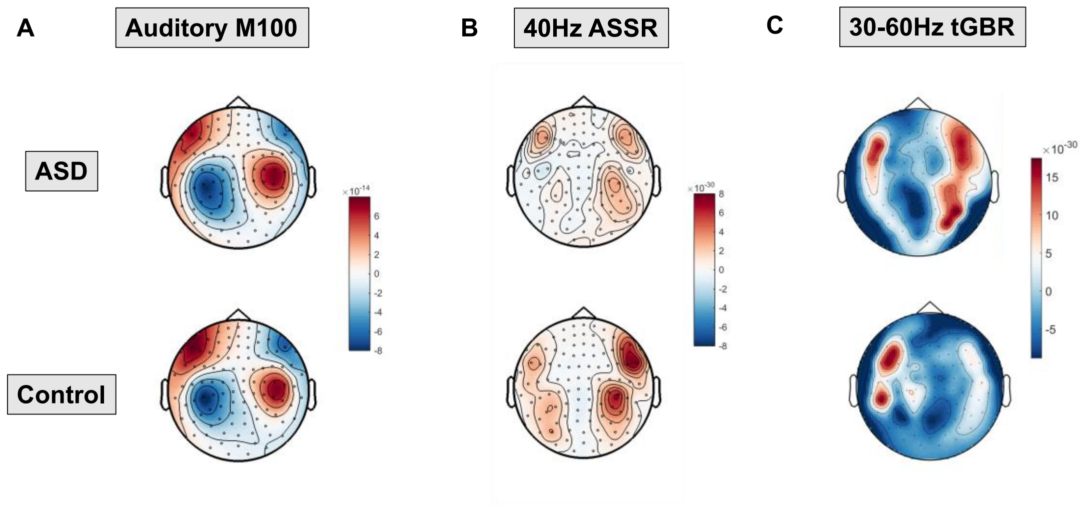

Supporting Figure 2: Sensor-level Analysis. **(A)** Group average topo-plot for the auditory M100 event-related field, magnetometers shown. **(B)** Group average topo-plot for auditory steady state responses (ASSR) at 40Hz **(C)** Group average topo-plot of the transient gamma-band response (tGBR), 30-60Hz, 0.0-0.1s. Scales represent MEG field strength, baseline-corrected, with units of Tesla/cm.

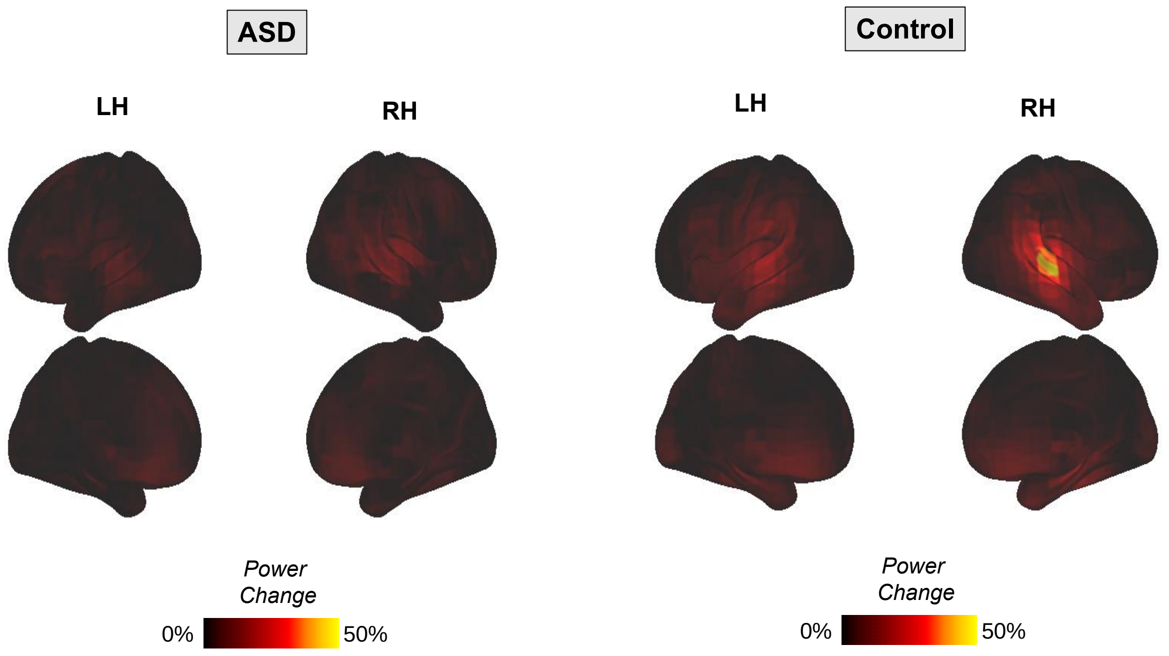

Supporting Figure 3: Whole-brain maps showing changes in ASSR power, corresponding to Figure 2 in the main text.

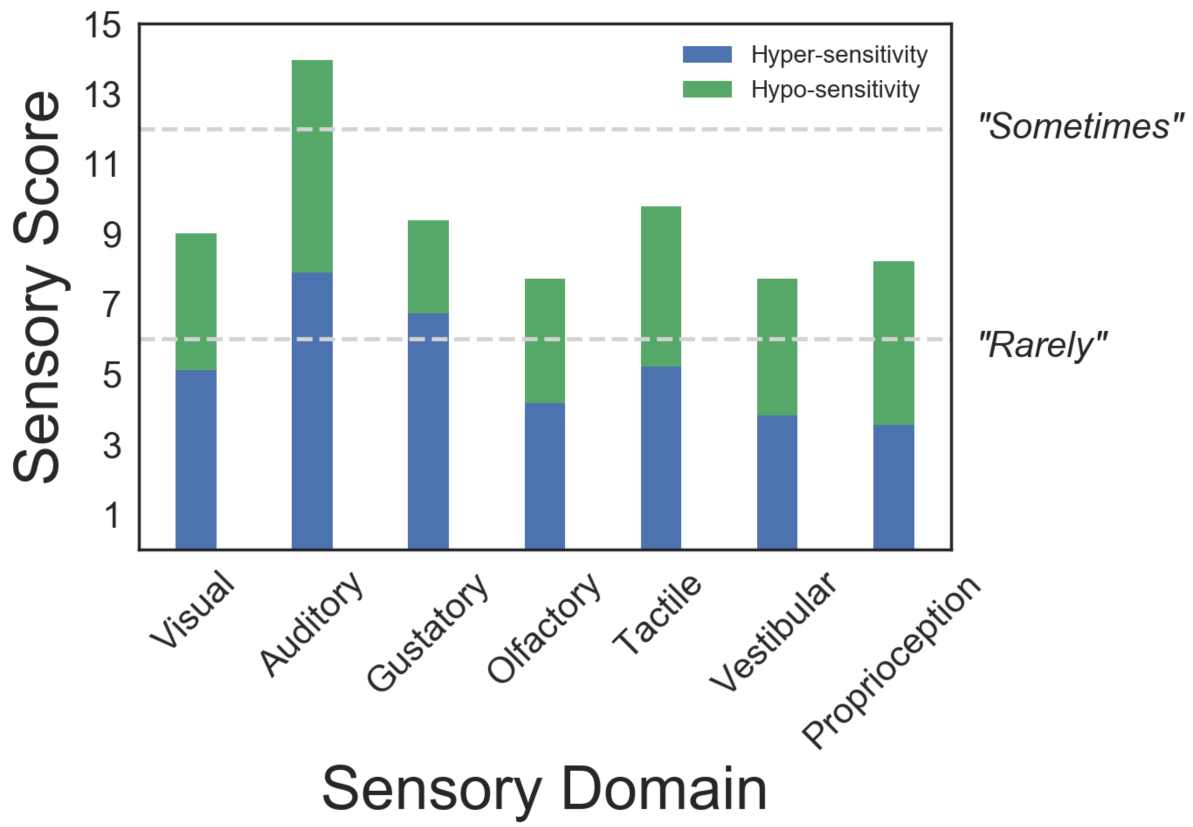

Supporting Figure 4: Responses to the Glasgow Sensory Questionnaire were grouped by sensory domain (maximum score = 20) and hypo- / hyper-sensitivity (green and blue bars respectively). Our data show a heterogeneous pattern of sensory symptoms, with mixture of hypo- and hyper-sensitivities. Auditory symptoms scored 13.9/20 corresponding to questionnaire answers closest to “Sometimes”.

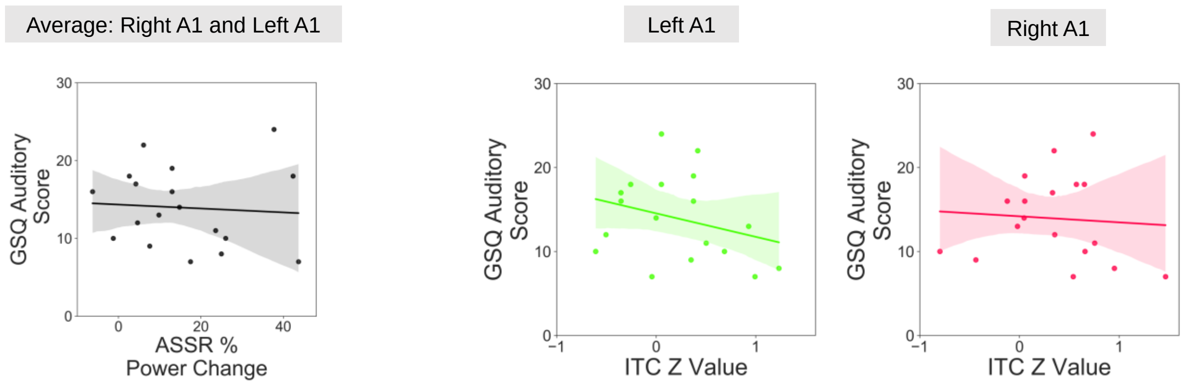

Supporting Figure 5: Scatter plots to show the relationship between ASSR power, averaged across left/right A1, and Glasgow Sensory Questionnaire (GSQ) Scores, summed across the six auditory questions only. There were no significant ($p>.05$) correlations for ASSR power, $r = -.07$, $p=.77$, or for ITC Z-Value (left A1), $r = -.29$, $p=.24$, (right A1), $r = -.07$, $p = .76$. The shaded region indicates 95% confidence intervals. ITC = Intertrial Coherence; ASSR = Auditory Steady State Response.

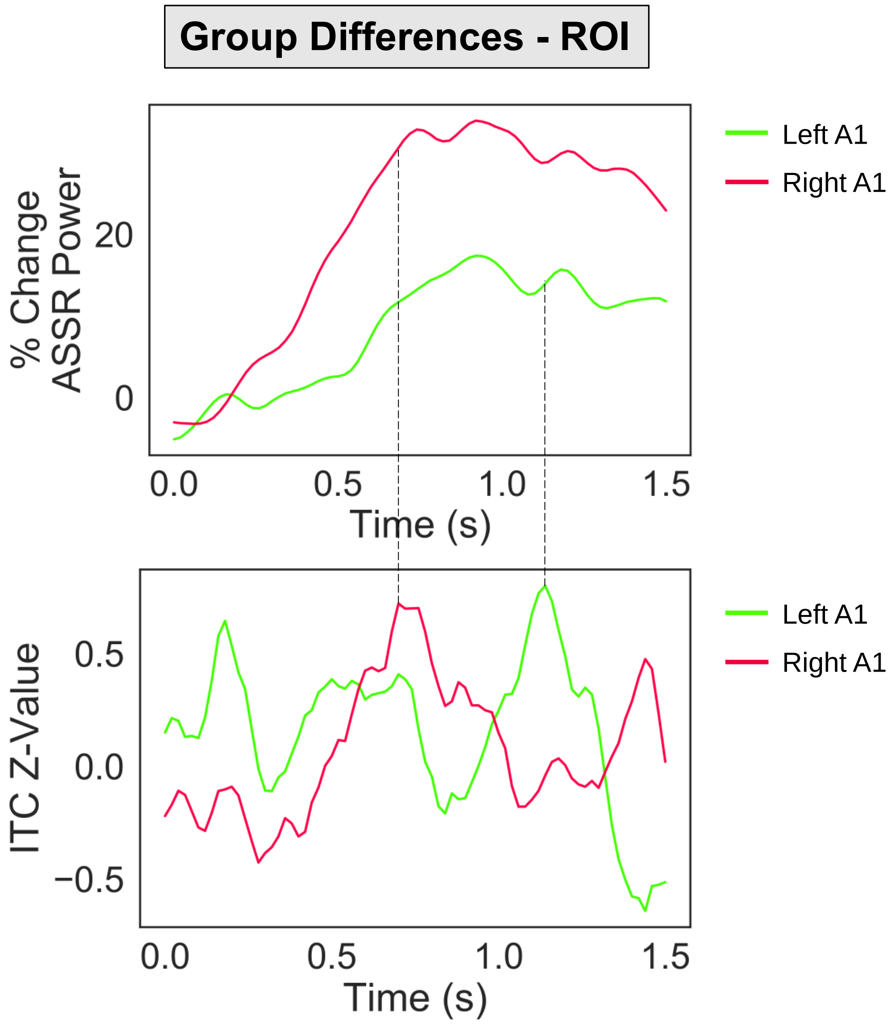

Supporting Figure 6: For ASSR power (top) and ITC power (bottom), the group average for the ASD group was subtracted from the group average for controls (at each time bin) and plotted for each ROI (right A1: red; left A1: green). The black dotted lines link the times maximum ITC group differences for left and right A1 with the corresponding data for ASSR power.

	Control		ASD	
Rank	*Search Term*	*Correlation*	*Search Term*	*Correlation*
1	auditory	0.635	auditory	0.471
2	superior temporal	0.595	auditory cortex	0.427
3	auditory cortex	0.57	superior temporal	0.424
4	sounds	0.567	sounds	0.408
5	listening	0.557	sound	0.404
6	sound	0.552	listening	0.398
7	temporal	0.55	heschl gyrus	0.392
8	planum temporale	0.539	heschl	0.39
9	temporale	0.539	primary auditory	0.389
10	planum	0.531	planum temporale	0.388

Supporting Table 1: Results of the meta-analytical decoding (top 10 terms shown). The correlation term corresponds to the r-value between the whole-brain unthresholded map and the Neurosynth concept-based meta-analysis maps, generated from over 10,000 neuroimaging studies.
